# Tenuifolin Attenuates Methamphetamine‐Induced Reinstatement in Mice by Regulating Hippocampal Postsynaptic BDNF Signaling

**DOI:** 10.1111/cns.70588

**Published:** 2025-08-28

**Authors:** Yize Qi, Shuyuan Fan, Yu Sun, Hanqing Shi, Hailing Li, Gang Xiao, Qingfeng Shen

**Affiliations:** ^1^ Institute for Stem Cell and Neural Regeneration and Key Laboratory of Cardiovascular &Cerebrovascular Medicine, School of Pharmacy Nanjing Medical University Nanjing Jiangsu China; ^2^ Department of Substance Dependence The Affiliated Xuzhou Eastern Hospital of Xuzhou Medical University, Xuzhou Eastern People's Hospital Xuzhou China; ^3^ Department of Anesthesiology Jiangsu Cancer Hospital, Jiangsu Institute of Cancer Research, The Affiliated Cancer Hospital of Nanjing Medical University Nanjing Jiangsu China

**Keywords:** BDNF, hippocampus, methamphetamine, PSD‐95, synaptic plasticity

## Abstract

**Background:**

Compulsive relapse (reinstatement) behavior of methamphetamine underlies the difficulty of withdrawal and is associated with abnormal BDNF‐mediated synaptic plasticity. However, how to intervene in this aberrant synaptic plasticity to prevent its reinstatement behavior in mice has not fully been studied.

**Methods:**

The CPP was used to establish a model of methamphetamine‐induced reinstatement behavior in C57BL/6 mice. Intraperitoneal injections of TEN were administered during the remission phase after the successful establishment of the CPP model to investigate the therapeutic effects on reinstatement. Immunofluorescence experiments were used to detect c‐fos expression in hippocampal CA1 neurons. Electrophysiological methods were used to determine glutamatergic transmission in hippocampal CA1 neural circuits. Western blotting was used to detect BDNF/TrKB and PSD‐95 protein expressions. Molecular docking was used to predict TEN molecule–protein binding.

**Results:**

Compared with control mice, METH‐treated mice presented increased CPP scores during the reinstatement phase, whereas, compared to METH‐treated mice, TEN‐treated mice presented significantly lower CPP scores. Immunofluorescence experiments indicated that TEN was able to inhibit the METH‐induced increase in c‐fos content. In addition, we found that TEN alleviates the METH‐triggered increase in glutamatergic transmission in mouse hippocampal CA1 neurons. Importantly, molecular docking studies demonstrated that TEN binds with BDNF, which may be important targets for its biological function. We also demonstrated that interfering with BDNF inhibits the therapeutic effect of TEN on the reinstatement of METH addiction.

**Conclusion:**

Our findings suggest that TEN treats METH‐induced reinstatement behavior by binding to BDNF, which may provide a novel target for treating relapse in patients addicted to METH.

## Graphical abstract

Tenuifolin attenuates methamphetamine‐induced reinstatement in mice by regulating hippocampal postsynaptic BDNF signaling. Left, The reduction of the expression of BDNF and PSD‐95 levels and abnormal synaptic plasticity promote METH‐induced reinstatement behavior in mice. Right, TEN treatment increases the expression of BDNF and PSD 95 and improves the function of synaptic plasticity to decrease METH‐induced reinstatement behavior.

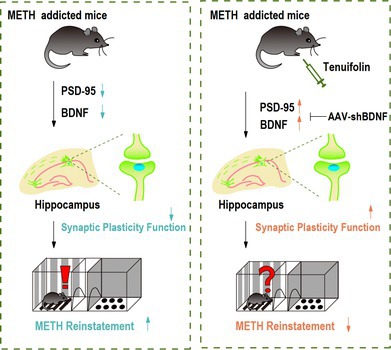

## 1 Introduction

Drug addiction is a chronic relapsing disorder that is believed to be aossociated with repeated drug exposure‐induced changes in the brain [1, 2, 3]. Methamphetamine (METH) is a potent central nervous system stimulant that has rewarding reinforcement [4]. An increasing number of studies have demonstrated that METH‐induced alterations in synaptic transmission are called “aberrant synaptic plasticity” [5, 6]. Therefore, targeting this aberrant synaptic plasticity may represent a strategy to treat METH addiction. Traditional Chinese Medicines have demonstrated significant potential in treating addiction, particularly in mitigating relapse behaviors driven by negative emotion [7, 8]. However, the specific mechanisms and active compounds responsible for these effects have not been fully identified. Exploring the relationship between the active components of Chinese herbs and the dysregulation of synaptic plasticity associated with methamphetamine addiction may offer novel therapeutic strategies for addressing drug‐seeking behaviors in METH users.

Addiction memory refers to the pathological memory formed through repeated exposure to addictive drugs, in association with cues or environments related to drug use [9, 10]. The persistence of this memory is central to the craving behavior and relapse observed in addicted individuals. It is widely accepted that once addiction memory is established, exposure to drug‐related cues triggers the retrieval of this memory, which subsequently induces craving and ultimately leads to relapse. Previous studies have demonstrated that the hippocampus plays a crucial role in mediating behaviors associated with drug reward [11, 12]. Drug‐induced abnormal synaptic plasticity is believed to be a key mechanism underlying addiction memory. Synaptic plasticity, which primarily consists of long‐term potentiation (LTP) and long‐term depression (LTD), serves as a fundamental model for understanding learned memory functions [13, 14, 15]. Alterations in synaptic plasticity in addiction are also characterized by dysregulation of the AMPAR‐to‐NMDAR ratio in addiction‐related brain regions [16, 17, 18]. Additionally, changes are accompanied by modifications in inter‐synaptic molecules, such as brain‐derived neurotrophic factor (BDNF) and postsynaptic density protein 95 (PSD‐95) [19, 20]. Studies have shown that BDNF expression has significantly decreased during the period following METH addiction, coinciding with impaired synaptic plasticity [21]. However, it remains unclear whether changes in BDNF levels during the METH addiction acquisition and relapse phases are directly associated with abnormal synaptic transmission during relapse. Given that BDNF contributes to these alterations in synaptic plasticity, BDNF may represent a promising target for the treatment of METH‐induced drug‐seeking behavior.

Tenuifolin (TEN) is a major bioactive component of the Chinese herb *Yuanzhi* (*Polygala tenuifolia*), renowned for its tranquilizing, anti‐inflammatory, and cognitive‐enhancing effects [22]. The pharmacokinetic properties of TEN facilitate its ability to cross the blood–brain barrier with ease, allowing for rapid distribution to brain tissues. Previous studies have demonstrated that TEN can improve the cognitive impairment induced by intrahippocampal injection of Aβ25‐35 in mice [23, 24]. Moreover, TEN ameliorates chronic restraint stress‐induced cognitive dysfunction by activating the BDNF/TrkB signaling pathway in the hippocampus [25]. However, whether TEN reduces aberrant synaptic plasticity and METH‐induced drug‐seeking behavior remains unclear.

In this study, we aimed to investigate whether TEN attenuates METH‐induced reinstatement and aberrant synaptic plasticity. Furthermore, we investigated the relationship between hippocampal postsynaptic BDNF and abnormal synaptic plasticity after repeated METH exposure. Finally, we investigated the restorative effect of TEN on BDNF in the hippocampus of METH‐administered mice and assessed its role in attenuating abnormal synaptic plasticity and METH‐induced seeking behavior.

## 2 Materials and Methods

### 2.1 Animals

C57BL/6 mice (male, 7–8 weeks old) were purchased from the Animal Core Facility of Nanjing Medical University. Animals were housed individually at 24°C ± 2°C and 55% ± 5% relative humidity. Food and water were provided ad libitum. The light–dark cycle was maintained at 12 h each (7:00 a.m. to 7:00 p.m.). All animal care and experimental procedures were approved by the Institutional Animal Care and Use Committee of Nanjing Medical University (IACUC‐2312045) and conducted in accordance with the Guiding Principles for the Care and Use of Laboratory Animals by the Chinese National Institute.

### 2.2 Chemicals and Regents

Tenuifolin (S9087) was purchased from Selleck (Selleck, Houston, USA). Monoclonal mouse GAPDH (60004‐1‐lg) and Polyclonal rabbit PSD‐95 (20665‐1‐AP) were purchased from Proteintech (Proteintech, Wuhan, China). Monoclonal mouse BDNF (A18129) was purchased from Abclonal (Abclonal, Wuhan, China). Monoclonal mouse c‐fos (ab208942) was purchased from Abcam (Abcam, Cambridge, UK). Anti‐mouse IgG (7076S) and Anti‐rabbit IgG (7074S) were purchased from CST (Cell Signaling Technology, Massachusetts, USA). rAAV‐hSyn‐mCherry‐5′miR‐30a‐shRNA(BDNF)‐3′miR‐30a (5.73 × 1012 vg/mL) was purchased from BrainVTA (BrainVTA, Wuhan, China). Reagents for electrophysiology were purchased from Sigma (Sigma‐Aldrich, Darmstadt, Germany).

### 2.3 Conditioned Place Preference

The Conditioned Place Preference (CPP) experiments were performed according to established protocols [5]. The CPP apparatus consisted of a three‐chamber design, with each lateral chamber exhibiting distinct visual and tactile characteristics. Behavioral testing employed C57BL/6 mice administered methamphetamine (METH, 2 mg/kg, i.p.) [26, 27]. On Day 0, initial preferences were determined by allowing the mice unrestricted exploration of the apparatus for 15 min. On Days 1, 3, 5, and 7, the mice received METH injections and were confined to the chamber opposite to their initial preference for 45 min. On Days 2, 4, 6, and 8, the mice received saline injections and were confined to their initially preferred chamber. Post‐testing was conducted on Day 9 for 15 min. From Day 10 to the extinction phase on Day 17, both saline and METH groups were received with a control solvent and were alternately confined to either chamber for 45 min daily. The treatment group received intraperitoneal injections of Tenuifolin (Selleck, USA, 20 or 40 mg/kg) following identical procedures. Extinction post‐test was assessed on Day 18 (15‐min), followed by a reinstatement test on Day 19. CPP behavior was quantified using Trackermaster software (Beijing, China).

### 2.4 Molecular Docking

The structures of the PSD‐95 protein were sourced from the Protein Data Bank (PDB) (https://www.rcsb.org/). The structures of the BDNF protein was predicted based on the protein amino sequence through AlphaFold Server (https://alphafoldserver.com/). Both structures were selected based on resolution and biological relevance to this study. The 3D structure of TEN was retrieved from the PubChem database (https://pubchem.ncbi.nlm.nih.gov/). Molecular Docking simulations were performed through the WeMol interface <Molecular Docking (AutoDock‐GPU v2)> [28].

### 2.5 Western Blotting

For Western blotting, mice were anesthetized with 5% isoflurane and euthanized by decapitation. Whole brains were rapidly excised, and hippocampal tissue was dissected on ice. Tissue samples were homogenized with RIPA buffer supplemented with 0.1 mM PMSF, incubated on ice for 30 min, followed by centrifugation at 12,000 × *g* for 15 min at 4°C. The supernatant was collected for protein quantification prior to denaturation. Proteins were resolved by SDS‐PAGE and transferred onto 0.45 μm PVDF membranes at 300 mA constant current for 60 min. Membranes were blocked with 7.5% nonfat milk in TBST for 1 h at room temperature, then incubated overnight at 4°C with primary antibodies. After three TBST washes, membranes were probed with HRP‐conjugated secondary antibodies for 1 h at room temperature. Protein bands were visualized using enhanced chemiluminescence and quantified with ImageJ software (v1.52a, NIH, USA) [16].

### 2.6 Histology

The histological examination procedure was as follows: Mice were anesthetized with 5% isoflurane and transcardially perfused with ice‐cold 4% paraformaldehyde (PFA) diluted in 1X phosphate‐buffered saline (PBS). Brains were postfixed in 4% PFA overnight at 4°C, dehydrated in 30% sucrose/PBS, then snap‐frozen in OCT compound. Coronal sections (30 μm) were obtained using a cryostat and rinsed in PBS. Sections were blocked with 5% serum for 1 h at room temperature. Sections were immunostained with primary antibodies overnight at 4°C, followed by species‐matched secondary antibodies for 2 h at room temperature. After DAPI nuclear counterstaining, sections were coverslipped using antifade mounting medium. Fluorescence images were acquired using a Nikon microscope. All images were processed using ImageJ software (v1.52a, USA).

### 2.7 Electrophysiology

Patch clamp electrophysiology experiments were conducted as previously described [15, 16, 29]. Briefly, the cutting solution consisted of the following components (in mM): 40 NaCl, 4.5 KCl, 1.25 NaH₂PO₄, 148.5 sucrose, 25 NaHCO₃, 0.5 CaCl₂, 7 MgSO₄, 10 glucose, 1 ascorbic acid, 3 sodium pyruvate, and 3 inositol, saturated with 95% O₂ and 5% CO₂. Coronal brain sections, 250 μm in thickness, were prepared from the hippocampal region using an oscillating slicer and incubated at 32°C for 45 min. The external solution contained (in mM): 1.25 NaH₂PO₄, 125 NaCl, 2.5 CaCl₂, 1.3 MgSO₄, 25 NaHCO₃, 4.5 KCl, 15 sucrose, and 15 glucose.

Whole‐cell recordings of spontaneous excitatory postsynaptic currents (sEPSCs) were performed using a cesium‐based internal solution containing (in mM): 119 CsMeSO₄, 5 QX‐314.Cl, 8 TEA.Cl, 15 HEPES, 0.6 EGTA, 0.3 Na₃GTP, 4 MgATP, and 7 phosphocreatine. AMPAR/NMDAR ratios and paired pulse ratios (PPRs) were recorded by inducing glutamatergic transmission in hippocampal CA1 neurons through electrical stimulation, with bipolar stimulating electrodes placed in the Schaffer collateral pathway delivering a stimulation that was 50 μs in duration and repeated at 20‐s intervals. PPRs of AMPAR‐mediated EPSCs were obtained by delivering two electrical stimulations 50 ms apart. AMPAR/NMDAR ratios were assessed by recording AMPAR‐mediated excitatory postsynaptic responses. Specifically, the AMPAR/NMDAR ratio was determined by recording the peak current of AMPAR‐mediated excitatory postsynaptic currents (EPSCs) at a holding potential of −70 mV, while the NMDAR‐mediated EPSCs were measured 50 ms after the AMPAR‐EPSC peak, at a holding potential of +40 mV. The AMPAR/NMDAR ratio was calculated by dividing the amplitude of the AMPAR‐EPSC by that of the NMDAR‐EPSC. For in vitro field potential recordings, the intra‐electrode solution contained 1 M NaCl. High frequency stimulation (HFS) was applied in three 100 Hz trains, each lasting 1 s with 30‐s intervals between trains. The slopes of the evoked fEPSPs were measured and normalized to the preconditioning baseline, allowing for the quantification of LTP magnitude.

### 2.8 Statistical Analysis

All experiments in this study were repeated at least three times and data are expressed as mean ± Standard Error for the Sample Mean (SEM). Electrophysiologic data were recorded using an IPA‐2 integrated patch amplifier controlled by SutterPatch software (Sutter Instrument, CA, USA). Statistical analyses were performed using one‐way ANOVA for comparisons between two or more groups, followed by Student–Newman–Keuls (SNK) multiple comparison test. Statistical calculations were performed using SigmaPlot. *p* < 0.05 was considered statistically significant.

## 3 Results

### 3.1 TEN Reduces the CPP Score and Hippocampal Neuronal Activity in METH‐Administrated Mice

**FIGURE 1:**
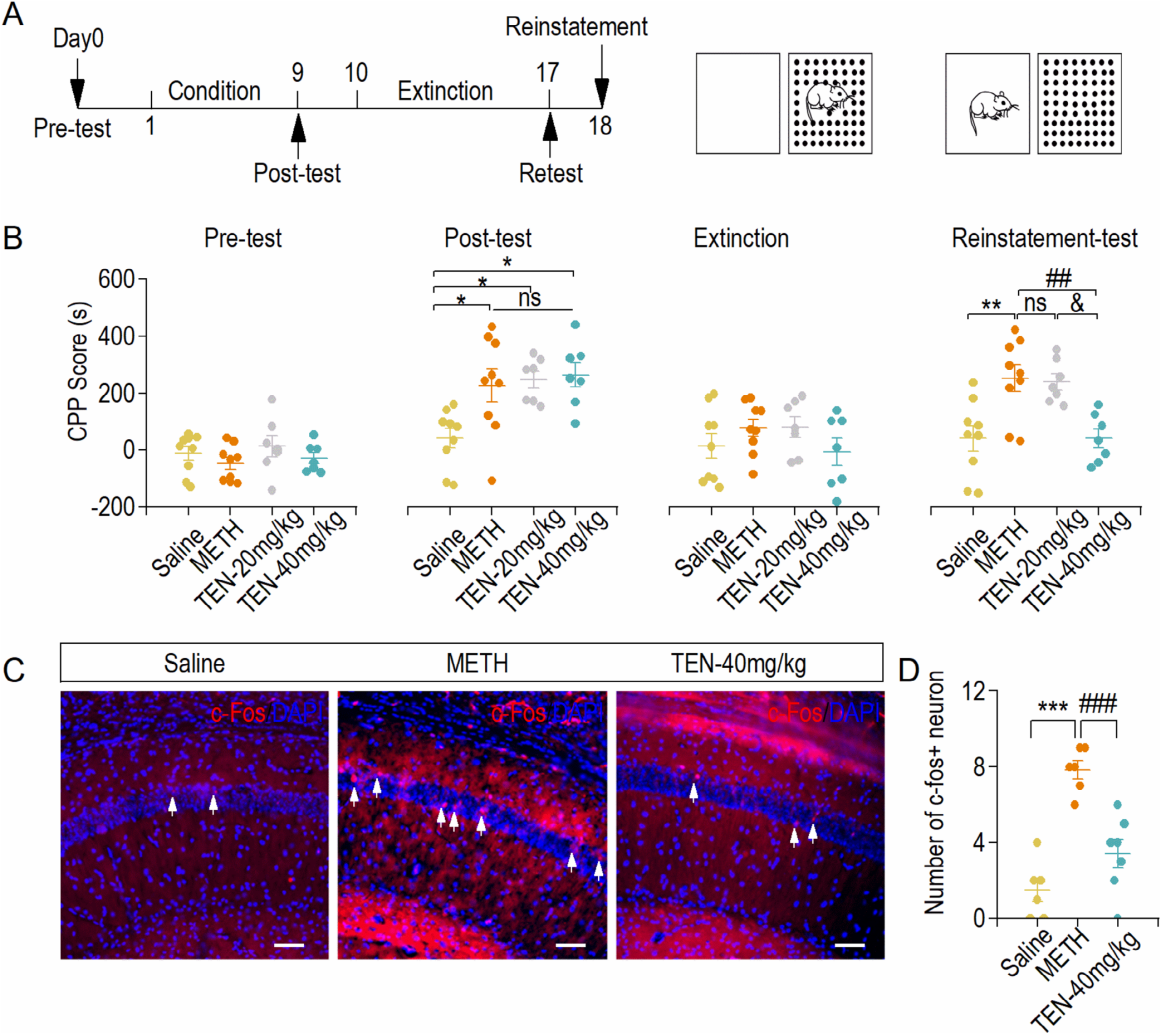
TEN reduces the CPP score and hippocampal neuronal activity in METH‐administrated mice. (A) Timeline of the CPP experiment. (B) CPP scores in the Saline, METH, TEN‐20 mg/kg, and TEN‐40 mg/kg groups during the pre, post, extinction, and reinstatement test periods. *p < 0.05 vs. the Saline group, **p < 0.01 vs. the Saline group, ^ns^ *p* > 0.05 vs. the METH group, ^##^ *p* < 0.01 vs. the METH group, and ^&^ *p* < 0.05 vs. the TEN‐20 mg/kg group, one‐way ANOVA, *n* = 9 mice (Saline and METH), 7 mice (TEN‐20 mg/kg and TEN‐40 mg/kg). (C) Sample c‐fos immunofluorescence images of hippocampal CA1 neurons from Saline, METH, and TEN‐40 mg/kg groups. Scale bar = 50 μm. (D) Statistics regarding the number of c‐fos^+^ cells. The number of c‐fos^+^ neurons in the TEN‐40 mg/kg group was lower than that in the METH group. ***p < 0.001 vs. the Saline group, ^###^ *p* < 0.001 vs. the METH group. SNK test with one‐way ANOVA, *n* = 6 slices from three mice (Saline and METH), *n* = 7 slices from three mice (TEN‐40 mg/kg).

To determine whether TEN plays a role in METH‐induced behaviors, we treated 8‐week‐old C57BL/6 mice with TEN during the extinction phase prior to the CPP resuscitation test (Figure 1A). During the resuscitation test phase, the CPP scores were significantly higher in the METH group compared with the saline group. Moreover, 20 mg/kg TEN administration had no significant effect on reducing CPP scores, whereas 40 mg/kg TEN decreased the Reinstatement‐test CPP score (Figure 1B). The hippocampus, particularly the CA1 region, plays a key role in drug addiction. The hippocampus also plays a role in drug withdrawal and relapse. After withdrawal, certain regions of the hippocampus may become more active, which may be associated with drug craving and relapse risk. Therefore, we examined the expression of c‐fos, a marker of hippocampal neuronal activity, in the three groups (Figure 1C). We found fewer c‐fos + cells in the METH + TEN group compared with the METH‐treated group (Figure 1D). These results suggest that TEN improved METH‐induced rescue behavior and decreased the activity of mouse hippocampal neurons.

### 3.2 TEN Treatment Reduces Methamphetamine‐Induced Potentiation of AMPAR‐Mediated Glutamatergic Transmission

**FIGURE 2:**
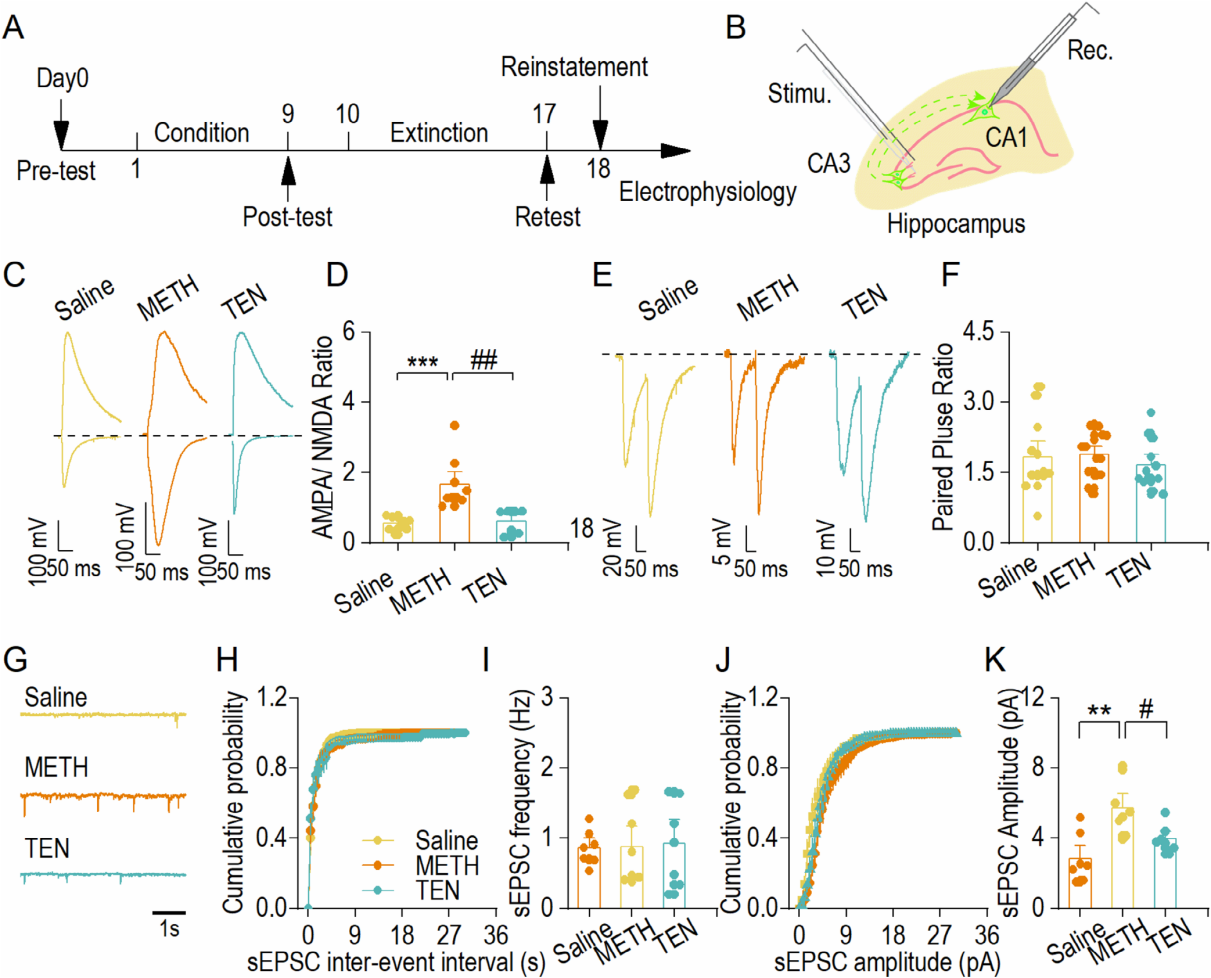
TEN treatment reduces methamphetamine‐induced potentiation of AMPAR‐mediated glutamatergic transmission. (A) Timeline diagram of the CPP and the whole‐cell electrophysiological experiments. (B) Schematic representation of electrical stimulation sites and recording locations for whole‐cell recording of hippocampal CA1 pyramidal neurons. (C) Sample traces of the AMPAR/NMDAR ratio in the Saline, METH, and TEN groups. (D) The bar chart shows a significant increase in the AMPAR/NMDAR ratio in the METH group compared with the Saline group. After treatment with TEN, this parameter returned to the levels observed in the Saline group. ***p < 0.001 vs. the Saline group, ^##^ *p* < 0.01 vs. the METH group. SNK test with one‐way ANOVA, *n* = 9 neurons from three mice (Saline and METH), *n* = 7 neurons from three mice (TEN). (E) Sample traces showing paired‐pulse ratios from the Saline, METH, and TEN groups. (F) The mean data of the bar graph show no difference in the PPR among the Saline, METH, and TEN groups. One‐way ANOVA, *n* = 12 neurons from three mice (Saline), *n* = 16 neurons from three mice (METH), and *n* = 12 neurons from three mice (TEN). (G) Sample traces showing sEPSCs from the Saline, METH, and TEN groups. (H) Plots graphs of sEPSCs frequency from Saline, METH, and TEN groups. (I) Summary bar graphs of sEPSCs frequency from Saline, METH, and TEN groups. One‐way ANOVA, *n* = 7 neurons from three mice (Saline), *n* = 8 neurons from three mice (METH), and *n* = 7 neurons from three mice (TEN). (J) Plots graphs of sEPSCs amplitude from the Saline, METH, and TEN groups. (K) Summary bar graphs of sEPSCs amplitude from the Saline, METH, and TEN groups. **p < 0.01 vs. the Saline group, ^#^ *p* < 0.05 vs. the METH group. One‐way ANOVA, *n* = 7 neurons from three mice (Saline), *n* = 8 neurons from three mice (METH), and *n* = 7 neurons from three mice (TEN). The data are presented as the means ± SEMs.

Previous studies have confirmed that the acquisition phase of methamphetamine addiction leads to abnormal increases in neuronal AMPAR‐mediated glutamatergic transmission [30]. To investigate whether the ameliorative effect of TEN on behavior occurs through AMPAR receptors, we performed membrane‐clamp electrophysiological experiments in C57BL/6 mice at the end of conditioned position preference experiments in the Saline group, the METH model group, and the 40 mg/kg TEN‐treated group (Figure 2A,B). We found that TEN significantly reduced the METH‐induced increase in the AMPAR/NMDAR ratio (Figure 2C,D). In contrast, no significant change in the PPR delivery ratio was observed (Figure 2E,F). Moreover, in the recordings of sPESCs, we found that the frequency of sEPSCs did not change significantly among the three groups, whereas the amplitude of the METH group was significantly increased compared to the Saline group, and the TEN group reduced the abnormal increase in the amplitude of the METH group (Figure 2G–K). In summary, TEN reduced the increase in AMPAR synapses in hippocampal CA1 neurons and alleviated the altered postsynaptic plasticity induced by METH.

### 3.3 Impairment of Synaptic Plasticity Is Prevented by TEN in METH‐Administrated Mice

**FIGURE 3:**
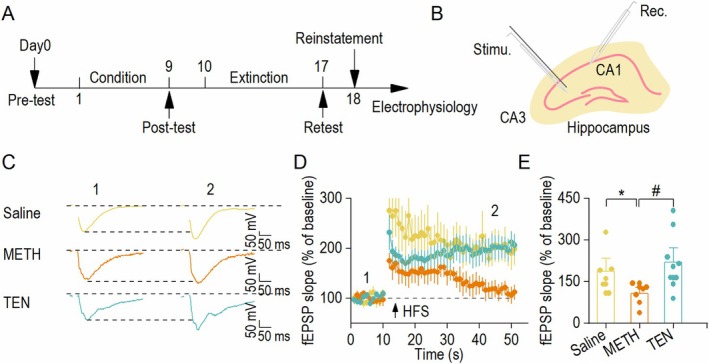
Impairment of synaptic plasticity is prevented by TEN in METH‐administrated mice. (A) Timeline of the CPP test and electrophysiological experiments of field EPSP recordings. (B) Schematic representation of the electrical stimulation site and recording location of CA1. (C) Sample traces showing the fEPSP in the three groups during baseline and after 30 to 40 min. (D) Summary of the timeline of the fEPSP slope in the three groups before, during, and after HFS induction. (E) Summary bar graphs of the fEPSP slope in the three groups. HFS did not elicit LTP in slices from the METH group but did elicit LTP in the Saline and TEN groups. *p < 0.05 vs. the Saline group, ^#^ *p* < 0.05 vs. the METH group, one‐way ANOVA, *n* = 6 slices from four mice (Saline), *n* = 7 slices from four mice (METH) and *n* = 9 slices from five mice (TEN). The data are presented as the means ± SEMs.

After confirming that TEN alleviates synaptic transmission and neuronal excitability abnormalities associated with METH addiction, we further investigated its effects on synaptic plasticity. At the conclusion of the behavioral studies, we used a field recording method to test high frequency stimulation (HFS)‐induced LTP in hippocampal CA1 neurons (Figure 3A,B). Compared with the METH group, the TEN group presented a significant reduction in the slope change within 10 min following HFS, indicating that TEN may have a therapeutic effect on METH‐induced LTP impairment (Figure 3C–E). These findings indicate that TEN prevents the METH‐induced impairment of synaptic plasticity in hippocampal CA1 neurons.

### 3.4 TEN Ameliorates METH‐Induced Hippocampal Synaptic Plasticity by Regulating PSD‐95 and BDNF Expression

**FIGURE 4:**
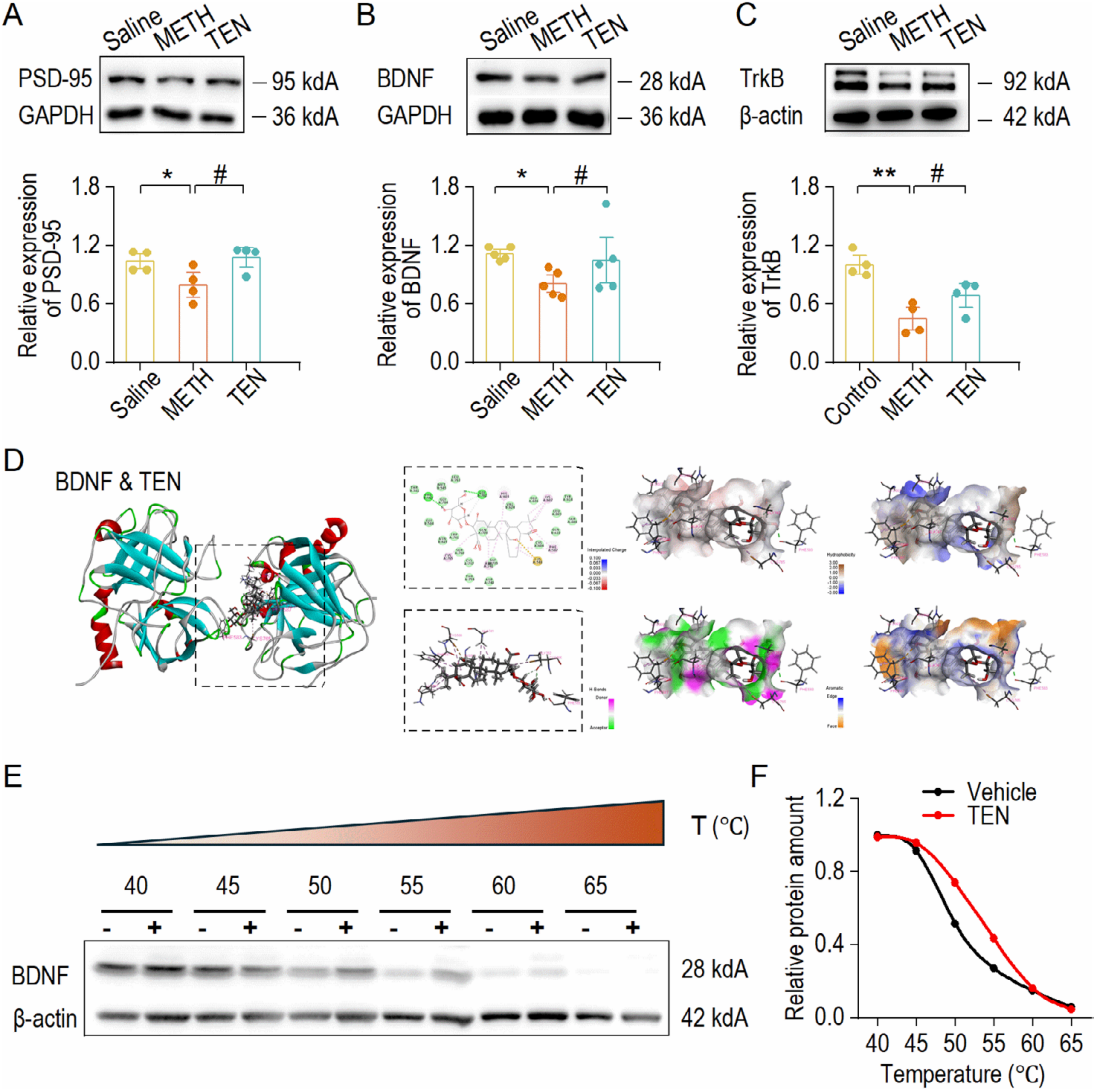
TEN ameliorates METH‐induced hippocampal synaptic plasticity by regulating PSD‐95 and BDNF expression. (A) Top, samples showing PSD‐95 expression in Saline, METH, and TEN groups. Bottom, the relative expression of PSD‐95 decreased in the METH group but increased after TEN treatment. *p < 0.05 vs. the Saline group, ^#^ *p* < 0.05 vs. the METH group. One‐way ANOVA. (B) Top, sample showing BDNF expression in the three groups. Bottom, the relative expression of BDNF decreased in the METH group but increased after TEN treatment. *p < 0.05 vs. the Saline group, ^#^ *p* < 0.05 vs. the METH group. One‐way ANOVA. (C) Top, samples showing the expression of TrkB in the three groups. Bottom, the relative expression of TrkB decreased in the METH group but increased after TEN treatment. **p < 0.01 vs. the Saline group, ^#^ *p* < 0.05 vs. the METH group, one‐way ANOVA. (D) Overall map of TEN docking with the BDNF molecule, 2D results of TEN docking with amino acid residues, and energy maps of the charge distribution and hydrogen bonding distribution between the TEN and surrounding amino acids. Binding energy = −7.6, RMSD = 27.87. (E) CETSA‐WB experiments confirmed the occurrence of binding between TEN and BDNF. (F) CETSA experiment showing the dissolution curves of BDNF protein in the solvent group and the TEN group at different temperatures.

METH impairs cognitive memory, a process linked to alterations in synaptic plasticity. These alterations are associated with the BDNF and PSD‐95 proteins. Using Western blotting, we confirmed that BDNF and PSD‐95 protein expression was reduced in the METH group compared to the saline group, whereas TEN treatment restored the expression of PSD‐95 and the BDNF/TrkB signaling pathway (Figure 4A–C). These findings indicate that TEN improves the expression of PSD‐95 and the BDNF/TrkB signaling pathway, suggesting that they contribute to the attenuation of functional synaptic plasticity. To explore whether TEN exerts its biological effects through interactions with BDNF, we employed large‐scale molecular docking models to predict its binding properties. The predictions indicated that TEN has binding affinity for BDNF (Figure 4D) and PSD‐95 (Figure S1). CETSA analysis of brain tissue revealed that, compared with the vehicle group, the TEN group presented increased thermal stability of BDNF (Figure 4E,F). This result aligns with molecular docking predictions, indicating that TEN directly binds to BDNF and PSD‐95. Consequently, TEN promotes BDNF expression and downstream signaling pathway activation.

### 3.5 Interference With BDNF Expression in the Hippocampal CA1 Region Reverses the Effect of TEN on Abnormal Synaptic Transmission in CA1 Neurons

**FIGURE 5:**
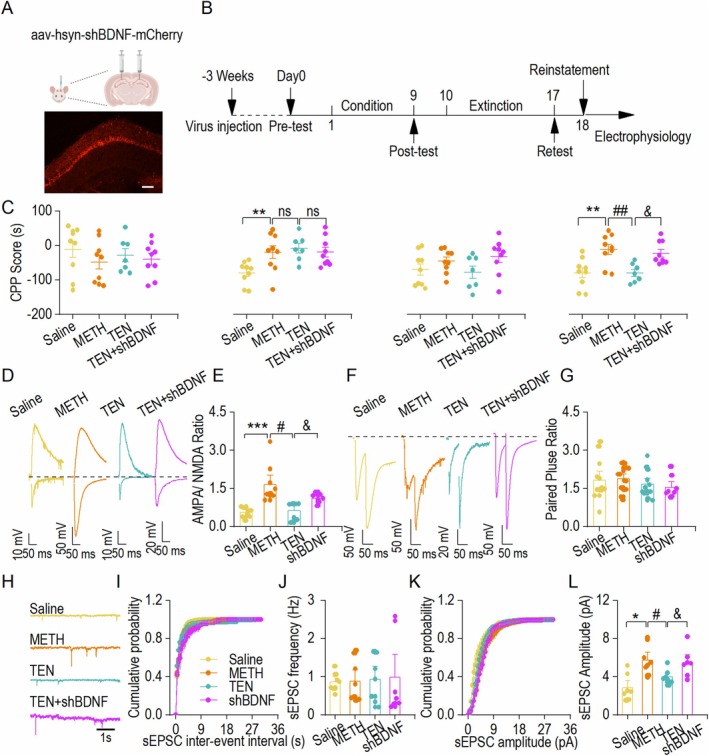
Interference with BDNF expression in the hippocampal CA1 region reverses the effect of TEN on abnormal synaptic transmission in CA1 neurons. (A) Schematic diagram of virus injection and sample image of virus expression in the hippocampus. (B) Timeline of the virus injection, CPP test, and whole‐cell electrophysiological experiments. (C) CPP scores in the four groups during the pre, post, extinction, and reinstatement test periods. **p < 0.01 vs. the Saline group, ^ns^ *p* > 0.05 vs. the Saline group, ^##^ *p* < 0.01 vs. the METH group, ^&^ *p* < 0.05 vs. the TEN group. One‐way ANOVA, *n* = 9 mice (Saline and METH), 7 mice (TEN), and 9 mice (shBDNF). The data from the Saline, METH, and TEN groups are references from Figure 1B. (D) Sample traces of the AMPAR/NMDAR ratio in the four groups. (E) Analysis of the NMDAR/AMPAR ratios. Bar graph statistics revealed that the AMPAR/NMDAR ratio was elevated in the METH group compared to the Saline group. Treatment with TEN restored this parameter to the level observed in the Saline group, whereas shBDNF attenuated the therapeutic effect of TEN. ***p < 0.001 vs. the Saline group, ^##^ *p* < 0.01 vs. the METH group, ^&^ *p* < 0.05 vs. the TEN group. SNK test with one‐way ANOVA; *n* = 9 neurons from three mice (Saline and METH), *n* = 7 neurons from three mice (TEN and shBDNF). The data from the Saline, METH, and TEN groups are references from Figure 2D. (F) Sample traces showing paired‐pulse ratios from the Saline, METH, TEN, and shBDNF groups. (G) Bar chart showing no significant differences in the PPR among the four groups. One‐way ANOVA, *n* = 12 neurons from three mice (Saline), *n* = 16 neurons from three mice (METH), *n* = 12 neurons from three mice (TEN), and *n* = 8 neurons from three mice (shBDNF). The data from the Saline, METH, and TEN groups are references from Figure 2F. (H) Sample traces showing sEPSCs from the four groups. (I) Plots graphs of sEPSCs frequency in the four groups. (J) Summary bar graphs of sEPSCs frequency in the four groups. Averaged data showing no significant differences among the four groups. One‐way ANOVA, *n* = 7 neurons from three mice (Saline), *n* = 8 neurons from three mice (METH), *n* = 7 neurons from three mice (TEN) and *n* = 7 neurons from three mice (shBDNF). The data from the Saline, METH, and TEN groups are references from Figure 2I. (K) Plots graphs of sEPSCs amplitude in the three groups. (L) Summary bar graphs of sEPSCs amplitude from three groups. *p < 0.05 vs. the Saline group; ^#^ *p* < 0.05 vs. the METH group; ^&^ *p* < 0.05 vs. the TEN group, one‐way ANOVA; *n* = 7 neurons from three mice (Saline), *n* = 8 neurons from three mice (METH) and *n* = 7 neurons from three mice (shBDNF). The data from the Saline, METH, and TEN groups are references from Figure 2K.

The results of previous experiments demonstrated that TEN binds to BDNF protein and affects its expression. To further verify whether TEN exerts its therapeutic effects through BDNF, we tested methamphetamine behavioral acquisition using WT mice that received bilateral hippocampal CA1 brain region injections of rAAV‐hSyn‐mCherry‐5'miR‐30a‐shRNA(bdnf)‐3'miR‐30a‐WPRE (shBDNF) administered during the extinction phase of TEN treatment (Figure 5A,B). After validation of shBDNF using immunofluorescence and Western blot methods (Figure S2 and Data S1), we tested the acquisition and reinstatement phases of CPP. We found that intervention with BDNF reversed the effects of TEN on CPP behavior in the reinstatement phase but not the acquisition phase, suggesting that TEN plays a role in reinstatement behavior via the BDNF signaling pathway (Figure 5C). At the end of the behavioral test, we recorded the AMPAR/NMDAR ratio, PPR and sEPSCs among the four groups using patch‐clamp electrophysiology, revealing that the AMPAR/NMDAR ratio was greater in the shBDNF+TEN group than in the TEN group (Figure 5D,E), In addition, there was no significant difference in the PPR (Figure 5F,G). The changes in sEPSCs frequency were not significantly different, but the amplitude was elevated (Figure 5H–L). The above results showed that interference with BDNF reversed the synaptic abnormality effect of TEN in improving CA1 neurons, indicating that TEN exerts its therapeutic effect through the BDNF pathway.

### 3.6 TEN‐Mediated Rescue of Impaired Synaptic Plasticity Is Inhibited Following the Downregulation of BDNF Expression in Hippocampal CA1 Neurons

**FIGURE 6:**
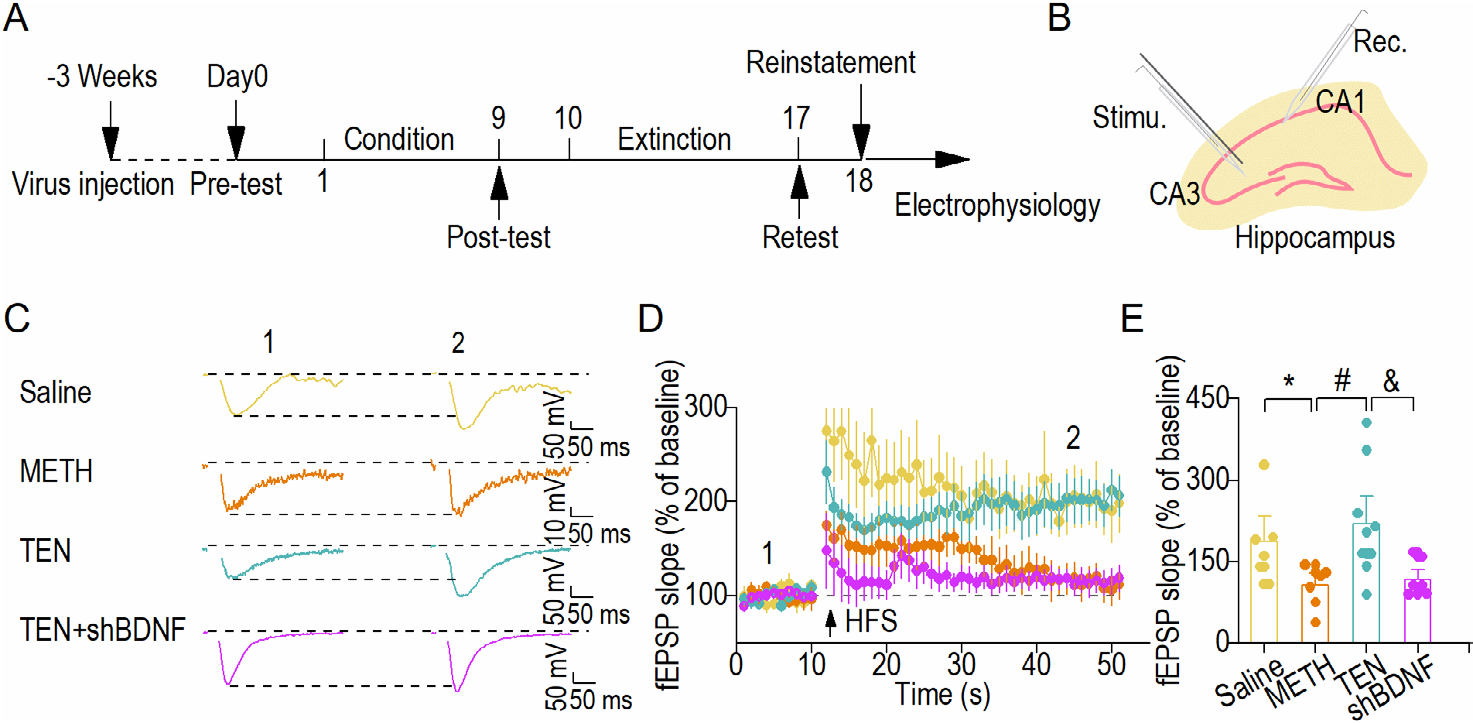
TEN‐mediated rescue of impaired synaptic plasticity is inhibited following the downregulation of BDNF expression in hippocampal CA1 neurons. (A) Timeline of the virus injection, CPP test, and field EPSP recordings. (B) Schematic representation of the electrical stimulation site and recording location of CA1. (C) Sample traces showing the fEPSP in the Saline, METH, TEN, and shBDNF groups at baseline and after 30 to 40 min. (D) Summary of the timeline of the fEPSP slope in the four groups before, during, and after HFS induction. (E) Summary bar graphs of the fEPSP slopes in the Saline, METH, TEN, and shBDNF groups. HFS did not elicit LTP in slices from the METH and TEN + shBDNF groups but did elicit LTP in the Saline and TEN groups. The data from the Saline, METH, and TEN groups are references from Figure 3D,E. *p < 0.05 vs. the Saline group, ^#^ *p* < 0.05 vs. the METH group, and ^&^ *p* < 0.05 vs. the TEN group; one‐way ANOVA, *n* = 6 slices from four mice (Saline), *n* = 7 slices from four mice (METH), *n* = 9 slices from five mice (TEN) and *n* = 7 slices from three mice (TEN+shBDNF).

After verifying that the injection of BDNF knockdown virus into the hippocampal CA1 brain region reverses the aberrant synaptic transmission and neuroprotective effects of TEN on METH‐induced abnormal synaptic transmission and neuroprotection in the hippocampal CA1, we further investigated the effects of BDNF knockdown on aberrant synaptic transmission and synaptic plasticity in the hippocampal CA1(Figure 6A,B). Hippocampal field potential tests were performed at the end of the behavioral studies. Compared with the TEN group, shBDNF + TEN group presented a significant decrease in the slope change in the latter 10 min after the induction of high frequency stimulation, suggesting that the therapeutic effect of TEN on the LTP damage caused by METH was significantly reduced after BDNF in the hippocampal CA1 was knocked down (Figure 6C–E). Together with the behavioral results, these data indicate that the ability of TEN to rescue impaired synaptic plasticity is prevented following the downregulation of BDNF expression in hippocampal CA1 neurons.

## 4 Discussion

In this study, we demonstrated that TEN attenuated CPP scores and decreased the activity of hippocampal CA1 neurons in the reinstatement phase in METH‐treated mice. Furthermore, TEN reduced the aberrant potentiation of glutamatergic transmission induced by METH administration. Although METH impairs synaptic plasticity and disrupts postsynaptic protein function, these effects are reversed following TEN treatments. Additionally, we found that TEN binds to BDNF molecules, alleviating METH‐induced reductions in BDNF/TrkB levels and correcting abnormalities in neuronal synaptic transmission. Notably, BDNF knockdown reversed the effects of TEN on behavior and synaptic plasticity, suggesting that TEN promotes increased BDNF expression by binding to BDNF, thereby preventing aberrant synaptic plasticity and reducing METH‐induced drug‐seeking behavior. Taken together, these findings suggest that TEN represents a potentially promising novel strategy to treat METH addiction.

The repeated use of METH can lead to the formation of addictive memories, which subsequently trigger drug‐seeking behavior [9, 31, 32]. Previous studies have characterized the molecular structure of TEN, noting its efficient transport across the blood–brain barrier, thereby influencing neuronal activity in the brain [22, 23]. Although TEN has positive effects on memory enhancement and sleep improvement in mouse models of Alzheimer's disease, it remains unclear how, or to what extent, TEN attenuates METH‐induced addictive memory and reinstatement behavior. We used TEN doses based on effective doses (40 mg/kg) reported in previous studies [33]. Our findings indicate that TEN reduces reinstatement behavior in METH‐administrated mice, suggesting a significant role for TEN in mitigating abnormal memory processes. Indeed, studies have shown that METH‐induced memory alterations contribute to drug‐seeking behavior [34, 35]. C‐fos, an immediate‐early gene, is commonly used as a marker of memory activity in the hippocampus [36, 37]. TEN was found to reduce METH‐induced changes in c‐fos expression, indicating that the attenuation of hippocampal activity by TEN may underlie its effects on reducing METH‐induced reinstatement behavior. Given that drug‐seeking behavior is associated with changes in hippocampal activity, the action of TEN may involve postsynaptic mechanisms. The abundance of AMPA receptors (AMPARs) in the postsynaptic region is closely related to LTP, LTD, and synaptic scaling [13, 38, 39]. Notably, a pathological increase in AMPAR levels was observed in hippocampal CA1 neurons of mice following METH addiction [5, 9, 40]. Our findings revealed that METH induced an increase in AMPAR‐mediated currents and an increase in the AMPAR/NMDAR ratio, which is consistent with previous reports suggesting that AMPAR is a major target affected by METH. Additionally, TEN reduced AMPAR‐mediated reactivity, suggesting that its effects are related to postsynaptic mechanisms impacting aberrant glutamatergic transmission. Indeed, aberrant synaptic plasticity may be mediated by calcium‐permeable AMPARs and normal synaptic plasticity may be compromised when glutamatergic transmission is pathologically elevated [41, 42]. We observed that exposure to METH reduced synaptic plasticity, and this effect was effectively reversed by TEN. Moreover, TEN treatment resulted in elevated PSD‐95 expression, further suggesting that TEN restores normal synaptic function.

Given that TEN restores synaptic plasticity, the mechanism underlying the role of TEN in METH‐induced drug‐seeking behavior needs to be identified. Previous studies have shown that METH inhibits the BDNF‐mediated signaling pathway and reduces protein levels in the mouse hippocampus [21, 43, 44]. Additionally, TEN alleviates Aβ protein‐induced decreases in BDNF and PSD‐95 expression levels in the HT‐22 cell line and ameliorates Aβ protein‐induced cognitive deficits in mouse models [24, 33, 45]. Therefore, TEN enhanced the expression of BDNF and PSD‐95 in METH‐exposed mice, suggesting that BDNF might be a key factor in TEN‐mediated attenuation of aberrant synaptic plasticity and behavior. Molecular docking analyses revealed that TEN binds to BDNF and PSD‐95, which may explain how TEN influences BDNF and PSD‐95. Therefore, we hypothesized that TEN increases BDNF and PSD‐95 levels via the BDNF pathway, thereby alleviating the METH‐induced synaptic abnormalities and reinstatement behavior. To test this hypothesis, the interference virus of BDNF was infused into the CA1 region to inhibit postsynaptic hippocampal CA1 neurons [46]. Behavioral assessments revealed that the BDNF interference virus prevents TEN attenuation of METH‐induced reinstatement behavior, suggesting that TEN prevents reinstatement behavior via a BDNF‐mediated mechanism. At the conclusion of the behavioral studies, whole‐cell patch‐clamp recordings and field potential measurements indicated that BDNF interference significantly reduced both the abnormal synaptic activity and the restoration of synaptic plasticity observed in TEN‐treated mice. These findings further suggest that the protective effects of TEN involve the modulation of synaptic activity via the BDNF pathway, potentially through interactions with the PSD‐95 [47, 48]. Thus, our results suggest that TEN ameliorates METH‐induced aberrant synaptic plasticity through the BDNF pathway, thereby attenuating METH‐induced reinstatement behavior.

## 5 Conclusions

In summary, we demonstrated that TEN reduces METH‐induced reinstatement behavior in mice. This effect is mediated through the prevention of aberrant synaptic plasticity, the restoration of impaired synaptic plasticity, and the restoration of reduced BDNF and PSD‐95 levels. Notably, the intervention of BDNF reversed the therapeutic effects of TEN on METH‐induced impairments in synaptic plasticity. Our findings suggest that TEN enhances BDNF expression and improves synaptic plasticity, demonstrating a potential strategy for the treatment of METH addiction.

## Author Contributions

Qingfeng Shen and Gang Xiao conceived and designed the study. Yize Qi and Shuyuan Fan performed the experiments. Yize Qi, Shuyuan Fan, and Yu Sun analyzed the data. Qingfeng Shen, Yize Qi, and Gang Xiao wrote the manuscript. Yu Sun, Hanqing Shi, and Hailing Li revised the manuscript. All authors have read and agreed to the published version of the manuscript.

## Conflicts of Interest

The authors declare no conflicts of interest.

## Supporting information


**Figure S1:** Tenuifolin binds to PSD‐95.
**Figure S2:** Validation of BDNF expression after injection of shBDNF virus.

**Data S1:** The uncropped gel/blot images for the expressions of proteins.

## Data Availability

The data that support the findings of this study are available from the corresponding author upon reasonable request.
