# Supplementary material for: Tenuifolin Attenuates Methamphetamine‐Induced Reinstatement in Mice by Regulating Hippocampal Postsynaptic BDNF Signaling
Source: CNS Neurosci Ther. 2025 Aug 28;31(8):e70588. doi: 10.1111/cns.70588 (PMC12391728; doi:10.1111/cns.70588)
Supplement: Supplementary file 1 — Figure S1: Tenuifolin binds to PSD‐95. Figure S2: Validation of BDNF expression after injection of shBDNF virus. [file CNS-31-e70588-s001.docx]

**Supplementary Figure 1** Tenuifolin binds to PSD-95. (A) Overall map of TEN docking with PSD-95 molecule, 2D results of TEN with amino acid residues, and energy maps of charge distribution and hydrogen bonding distribution between TEN and surrounding amino acids.





**Supplementary Figure 2** Validation of BDNF expression after injection of shBDNF virus. (A) Sample images of BDNF immunofluorescence and of hippocampal CA1 neurons from sh-NC and shBDNF groups. Scale bar = 100 μm. (B) Top, Sample’s showing the expression of BDNF in sh-NC and shBDNF groups. Bottom, the relative expression of BDNF decreased in the shBDNF group. **p* < 0.05 vs. the sh-NC group. One-way ANOVA. (C) Top, Sample’s showing the expression of TrkB in sh-NC and shBDNF groups. Bottom, the relative expression of BDNF decreased in the shBDNF group. **p* < 0.05 vs. the sh-NC group. One-way ANOVA.
