# Supplementary material for: Tenuifolin Attenuates Methamphetamine‐Induced Reinstatement in Mice by Regulating Hippocampal Postsynaptic BDNF Signaling
Source: CNS Neurosci Ther. 2025 Aug 28;31(8):e70588. doi: 10.1111/cns.70588 (PMC12391728; doi:10.1111/cns.70588)

A

Full unedited gel/blot for Figure 4A and B

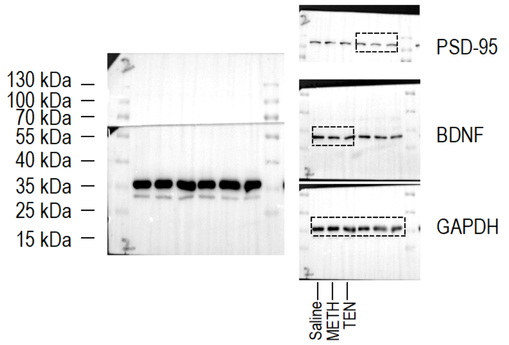


B

Full unedited gel/blot for Figure 4C

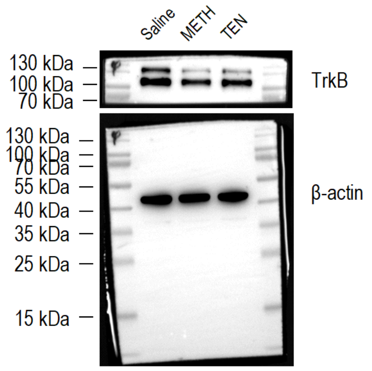


C

Full unedited gel/blot for Figure 4E

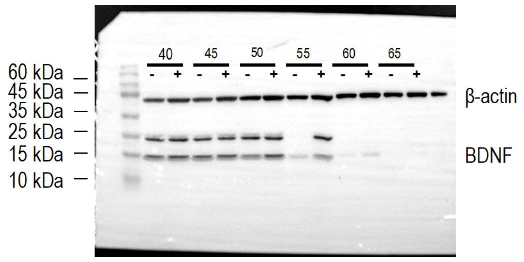


D

Full unedited gel/blot for Figure S1B

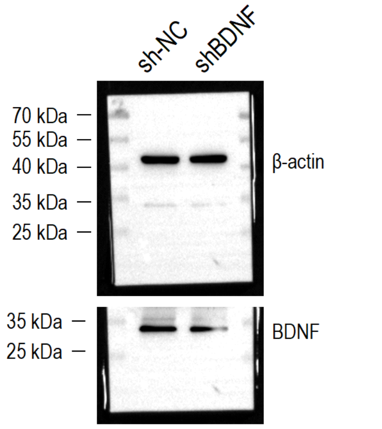


E

Full unedited gel/blot for Figure S1C

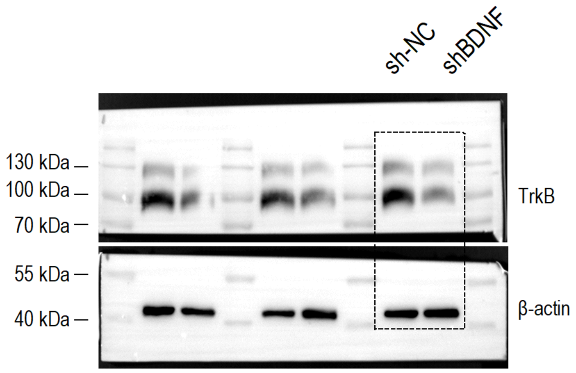

Supplement: Supplementary file 2 — Data S1: The uncropped gel/blot images for the expressions of proteins. [file CNS-31-e70588-s002.pdf]
